# Metabolomic Analysis to Elucidate Mechanisms of Sunitinib Resistance in Renal Cell Carcinoma

**DOI:** 10.3390/metabo11010001

**Published:** 2020-12-22

**Authors:** Tomonori Sato, Yoshihide Kawasaki, Masamitsu Maekawa, Shinya Takasaki, Kento Morozumi, Masahiko Sato, Shuichi Shimada, Naoki Kawamorita, Shinichi Yamashita, Koji Mitsuzuka, Nariyasu Mano, Akihiro Ito

**Affiliations:** 1 Department of Urology, Tohoku University Graduate School of Medicine, Sendai, Miyagi 980-8574, Japan; tomonori4659@uro.med.tohoku.ac.jp (T.S.); kenmorozm@gmail.com (K.M.); masahiko@uro.med.tohoku.ac.jp (M.S.); shimapp@uro.med.tohoku.ac.jp (S.S.); kawamoritan@gmail.com (N.K.); yamashita@uro.med.tohoku.ac.jp (S.Y.); mitsuzuka@uro.med.tohoku.ac.jp (K.M.); itoaki@uro.med.tohoku.ac.jp (A.I.); 2 Department of Pharmaceutical Sciences, Tohoku University Hospital, Sendai, Miyagi 980-8574, Japan; masamitsu.maekawa.a2@tohoku.ac.jp (M.M.); takasaki_shinya@hosp.tohoku.ac.jp (S.T.); mano@hosp.tohoku.ac.jp (N.M.)

**Keywords:** metabolomics, resistance, renal cell carcinoma, sunitinib, glutamine

## Abstract

Metabolomics analysis possibly identifies new therapeutic targets in treatment resistance by measuring changes in metabolites accompanying cancer progression. We previously conducted a global metabolomics (G-Met) study of renal cell carcinoma (RCC) and identified metabolites that may be involved in sunitinib resistance in RCC. Here, we aimed to elucidate possible mechanisms of sunitinib resistance in RCC through intracellular metabolites. We established sunitinib-resistant and control RCC cell lines from tumor tissues of RCC cell (786-O)-injected mice. We also quantified characteristic metabolites identified in our G-Met study to compare intracellular metabolism between the two cell lines using liquid chromatography-mass spectrometry. The established sunitinib-resistant RCC cell line demonstrated significantly desuppressed protein kinase B (Akt) and mesenchymal-to-epithelial transition (MET) phosphorylation compared with the control RCC cell line under sunitinib exposure. Among identified metabolites, glutamine, glutamic acid, and α-KG (involved in glutamine uptake into the tricarboxylic acid (TCA) cycle for energy metabolism); fructose 6-phosphate, D-sedoheptulose 7-phosphate, and glucose 1-phosphate (involved in increased glycolysis and its intermediate metabolites); and glutathione and myoinositol (antioxidant effects) were significantly increased in the sunitinib-resistant RCC cell line. Particularly, glutamine transporter (SLC1A5) expression was significantly increased in sunitinib-resistant RCC cells compared with control cells. In this study, we demonstrated energy metabolism with glutamine uptake and glycolysis upregulation, as well as antioxidant activity, was also associated with sunitinib resistance in RCC cells.

## 1. Introduction

Renal cell carcinoma (RCC) is a urological malignancy, and its incidence has steadily increased annually [1,2]. Several small-molecular agents that inhibit proangiogenic receptor tyrosine kinases, such as antiangiogenic multityrosine kinase inhibitors (TKIs) like sunitinib, pazopanib, or axitinib, have been widely used for patients with metastatic or recurrent RCC [3]. Sunitinib is a multikinase inhibitor that targets vascular endothelium-derived VEGFR-1–3, PDGFR-α/-β, and fetal liver tyrosine kinase receptor and inhibits downstream signals such as protein kinase B (Akt) and mammalian target of rapamycin (mTOR) [4,5]. Although several mechanisms of acquired TKI resistance including P-glycoprotein expression and associations with microRNA have been reported, therapeutic strategies for these resistance cases have not yet been established [6,7,8].

Metabolomics is a comprehensive analysis that measures the wide range of metabolites, allowing for real-time quantification of changes in cellular metabolism [9]. Cancer metabolism has been shown to utilize the Warburg effect (aerobic glycolysis), and intermediate metabolites affect cellular metabolism, cell proliferation, and immunosuppression [10]. Measurement of metabolite upregulation accompanying cancer progression may be a promising technique for discovery of therapeutic targets [11]. Several studies on the metabolites of RCC have been reported [12,13,14]. Although a previous report investigated the effect of chemotherapy on the metabolic profile, the relation between sunitinib resistance in RCC and cellular metabolism has not been fully elucidated [15].

We previously investigated metabolites of clear cell RCC (ccRCC) by using our global metabolomics (G-Met) protocol in tissue samples [16,17]. Pathways of metabolites such as glutathione, tryptophan and glycolysis were associated with diagnosis and malignant status. Moreover, glycoglycerolipid, carnitine, and tocopherol pathways were associated with diagnosis, while TCA cycle, nucleotide sugar, and inositol pathways were associated with malignant status [16]. Cancer metabolism is thought to be regulated by various signaling pathways, such as Akt activity, which affects aerobic glycolysis and mitochondrial function downregulation [18,19,20]. Recently, mesenchymal-to-epithelial transition (MET) receptors were associated with sunitinib resistance in xenograft models of RCC [21]. However, the association between cancer metabolism involved in sunitinib resistance must be clarified.

In the present study, we constructed a precise quantitative measurement system using liquid chromatography-mass spectrometry (LC-MS) to evaluate intracellular metabolites of sunitinib resistance among characteristic metabolites considered to contribute to sunitinib resistance.

## 2. Results

### 2.1. Comparison of Growth Inhibitory Effect of Sunitinib in Cell Lines

To evaluate the growth inhibitory effect of sunitinib on 786-O, ACHN, and Caki-1 cell lines, water-soluble tetrazolium salt (WST) assay was performed to compare 50% cell growth inhibitory concentrations (IC_50_). IC_50_ values were as follows: 786-O: 4.6 µM [95% confidence interval (CI) 1.1–18.4], ACHN: 1.9 µM (95% CI 0.75–5.9), and Caki-1: 2.8 µM (95% CI 0.6–12.7). 786-O had a higher IC_50_ value than the other two cell lines, and only 786-O could be stably subcultured using the previously reported sunitinib-resistant cell preparation protocol [22,23,24]. Therefore, 786-O was used for further study.

### 2.2. Establishment of a Sunitinib-Resistant 786-O Cell Line (786-R) for In Vitro and In Vivo Examination of Sunitinib Resistance

**Figure 1:**
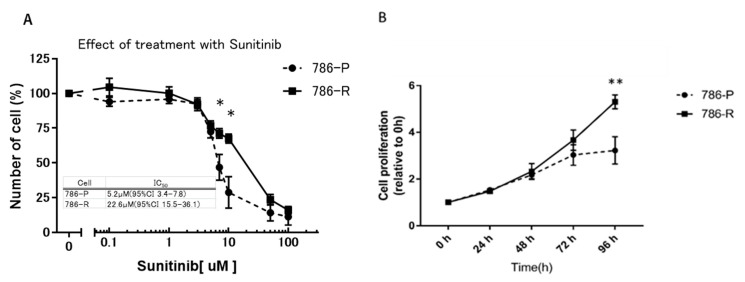
Cellular profile of established 786-R cells in vitro: (**A**) Effect of sunitinib treatment between 786-P and 786-R cells in vitro. Sunitinib-resistant cell line 786-O (786-R) and the parental cell line 786-O (786-P) were treated with sunitinib at indicated concentrations. Data are shown as mean ± standard error of the mean. * *p* < 0.05, ** *p* < 0.01. (**B**) Cell proliferation under sunitinib exposure. Significant enhancement of 786-R cell proliferation was observed at 96 h after sunitinib (5 µM) exposure compared with that in 786-P cells. All experiments were repeated in triplicate in three independent experiments. Data are shown as mean ± standard deviation. Data were analyzed for statistical significance by the Mann–Whitney U test. * *p* < 0.05, ** *p* < 0.01.

786-O was passaged for at least 20 passages in medium containing 10 μM sunitinib, and finally the sunitinib-resistant 786-O cell line (786-R) was established in vitro. WST assay was performed to compare sunitinib sensitivities of parent strain (786-P) and resistant strain 786-R. IC_50_ values were as follows: 786-P: 5.2 µM (95% CI 3.4–7.8) and 786-R: 22.6 µM (95% CI 15.5–36.1); 786-R showed about 4.3-fold drug resistance (*p* < 0.05) (Figure 1A). Next, 786-P and 786-R were cultured under 5 µM sunitinib to evaluate proliferation ability. At 96 h after sunitinib exposure, 786-R demonstrated significantly increased proliferative ability compared with 786-P (*p* < 0.01) (Figure 1B).

**Figure 2:**
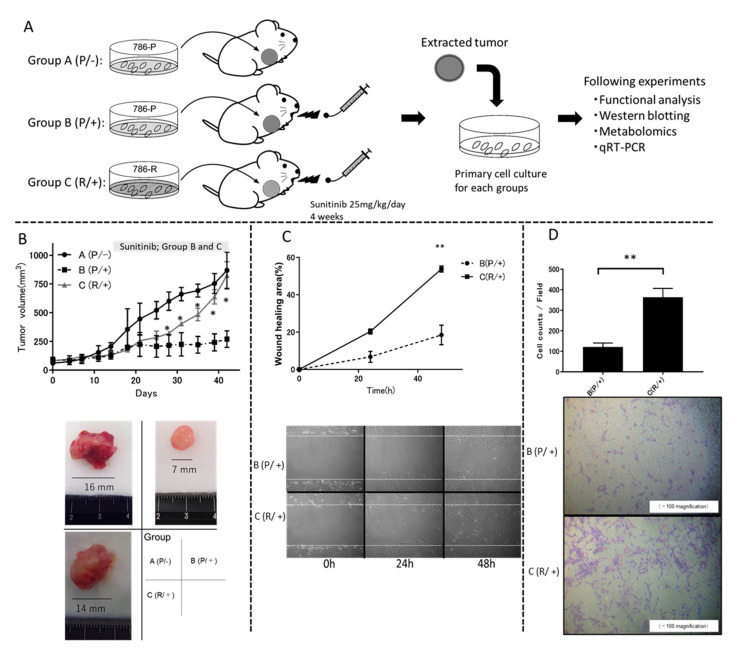
Establishment of sunitinib-resistant cells and cell profiles: (**A**) Three experimental mouse groups were created (n = 5/group): group A (P/−), 2 × 10^6^ 786-P cells were transplanted and sunitinib was not orally administered; group B (P/+), 2 × 10^6^ 786-P cells were transplanted and sunitinib was orally administered; and group C (R/+), 2 × 10^6^ 786-R cells were transplanted and sunitinib was orally administered. Sunitinib dose was 25 mg/kg/day. (**B**) Group C (R/+) showed a significant increase in tumor volume compared with group B (P/+) after 15 days of sunitinib treatment. Tumor volume was calculated using the modified ellipsoid formula 1/2 (length × width^2^) after transplantation. (**C**). In the wound healing assay, the migration area was calculated every 24 h under exposure to sunitinib. The ratio of the migration area to the scratch area was graphed. Group C (R/+) showed increased migration ability compared with group B (P/+) at 48 h after sunitinib exposure (*p* = 0.003). The experiment was carried out in triplicate and repeated three times. (**D**) In the two-chamber assay, group C (R/+) demonstrated significantly increased invasion ability under sunitinib exposure compared with group B (P+). The cells that invaded through the membrane to the lower surface were counted. The experiment was carried out in triplicate and repeated three times. Data of (**B**–**D**) are shown as mean ± standard deviation. Data of (**B**–**D**) were analyzed for statistical significance by the Mann–Whitney U test. * *p* < 0.05 ** *p* < 0.01.

786-P or 786-R was subcutaneously implanted into BALB/c-nu/nu mice, and the effects of sunitinib administration on tumor volume were compared (Figure 2A,B). There was a significant difference in tumor volume between group B (P/+) and group C (R/+) from day 15 after sunitinib administration (Figure 2B). Tumor tissues excised from each group were subjected to primary cell culture and used for further studies. We next compared the migration ability and invasion ability of primary cultured cells from group B (P/+) and C (R/+) subcutaneous tumors obtained in vivo with and without sunitinib exposure for subsequent in vitro assay. In the wound healing assay, there was no difference in the migration area between group B (P/+) and group C (R/+) without exposure to sunitinib (data not shown). Conversely, under sunitinib exposure, the migration area was significantly increased in group C (R/+) compared with that in group B (P/+) (*p* < 0.01) (Figure 2C). In the two-chamber assay, there was no difference in the number of infiltrating cells between group B (P/+) and group C (R/+) without exposure to sunitinib (data not shown). Conversely, under sunitinib exposure, the invasive ability was significantly increased in group C (R/+) compared with group B (P/+) (363 ± 14.5 cells/field vs. 121.1 ± 6.4 cells/field, respectively, *p* < 0.01) (Figure 2D).

**Figure 3:**
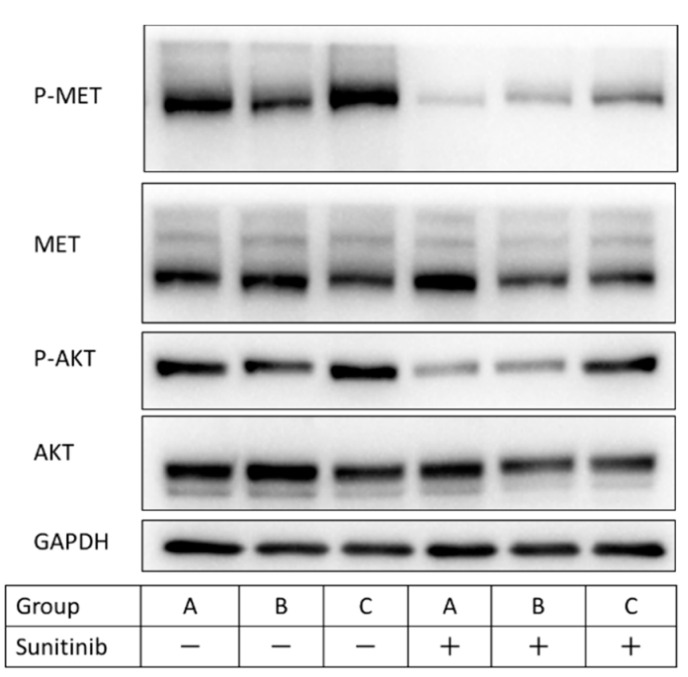
Comparison of the activation pattern of the signal transduction system in sunitinib-resistant cells by western blotting. Group A (P/−) was subcutaneously transplanted with 786-P and did not receive sunitinib, group B (P/+) was subcutaneously transplanted with 786-P and received sunitinib treatment, and group C (R/+) was subcutaneously transplanted with 786-R and received sunitinib treatment in vivo. Primary cultured cells were obtained from these mouse models, the cells in each group were cultured in medium with or without sunitinib 5 µM for 48 h. In a series of western blot analyses, at least three independent experiments were performed with similar results.

Western blotting was performed to confirm Akt and MET phosphorylation in group C (R/+) because of their association with sunitinib resistance. Changes in the activation pattern of signal transduction (Akt, MET) in the presence and absence of sunitinib of three groups of primary cultured cells obtained in vivo were examined by western blotting. In the absence of sunitinib, Akt and MET phosphorylation was confirmed in all groups. However, in the presence of sunitinib, significant MET phosphorylation was observed in group C (R/+) compared with that in group A (P/−) (*p* = 0.043) and tended to be increased between group C (R/+) and group B (P/+) (*p* = 0.064). Akt phosphorylation in the presence of sunitinib was suppressed in groups A (P/−) and B (P/+) and was significantly desuppressed in group C (R/+) compared with the other groups (*p* < 0.05) (Figure 3). From these results, it was confirmed that sunitinib-resistant cells could be established in vitro and in vivo.

### 2.3. Identification of Upregulated Metabolites in Sunitinib-Resistant Cells

**Figure 4:**
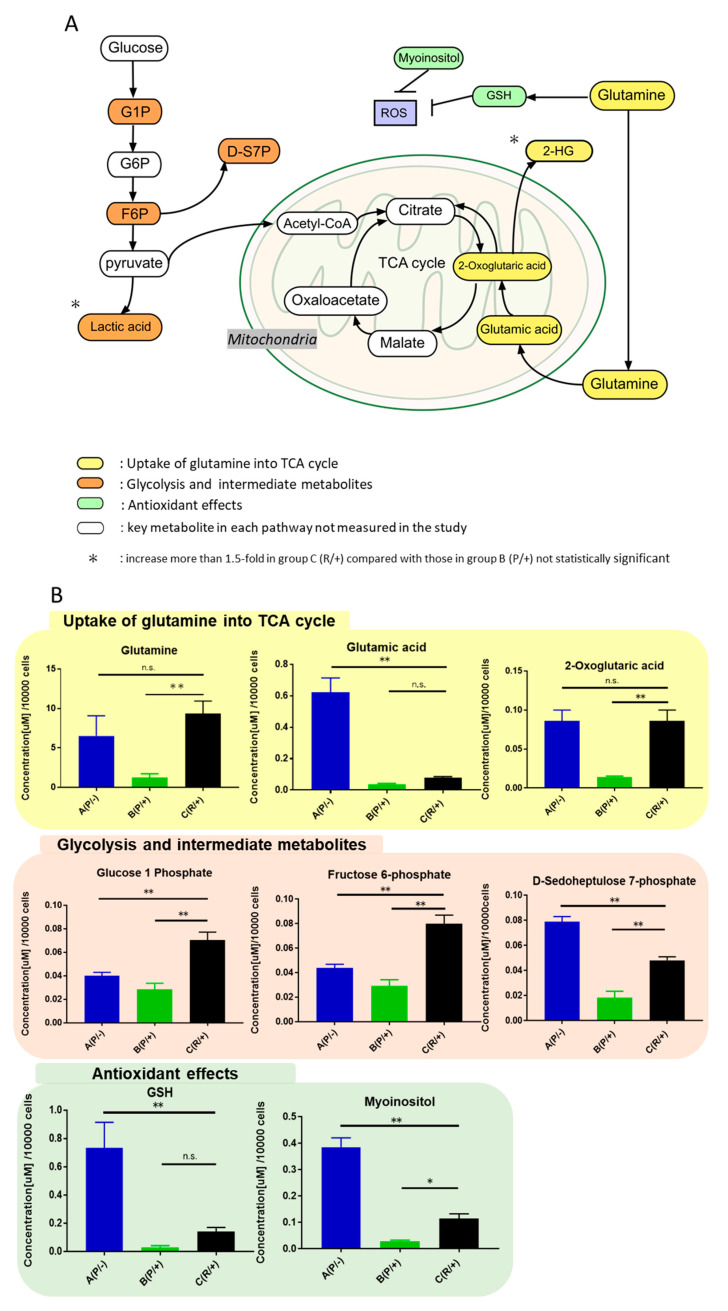
Metabolites increased in sunitinib-resistant cells and their metabolomic pathways. (**A**) Eleven metabolites were significantly increased in group C (R/+) compared with group B (P/+) (*p* < 0.05 for all). Among these increased metabolites, we focused eight metabolites that glutamine, glutamic acid, and 2-oxoglutaric acid were grouped in the glutamine pathway (yellow), an increase in glycolysis and its intermediate metabolites (fructose 6-phosphate, d-sedoheptulose 7-phosphate, and glucose 1-phosphate) (orange) was indicated, and glutathione and myoinositol (green) have antioxidant effects against reactive oxidative species (ROS). Lactic acid and 2-HG tended to increase more than 1.5-fold in group C (R/+) compared with those in group B (P/+) not statistically significant. (added an asterisk). Although not measured, key metabolites that constitute metabolic pathways were inserted (white). (**B**) Metabolites elevated in sunitinib-resistant cells were compared between the three groups including control group A (P/−) without sunitinib exposure. Glutamic acid, glutathione (GSH), and myoinositol levels in sunitinib-resistant cells [group C (R/+)] with sunitinib exposure were different than those in the control group A (P/−). However, most metabolites in group C (R/+) recovered to greater than the respective levels in group A (P/−). The obtained intracellular metabolite concentration was counted for each cell, and the concentration per 10,000 cells was calculated. Cells were cultured in triplicate, and measurements were performed three times to calculate the mean metabolite concentration. Differences in experimental groups were determined by one-way ANOVA followed by the post hoc Tukey’s test. * *p* < 0.05, ** *p* < 0.01.

To identify the characteristic metabolites increased in group C (R/+) under sunitinib-resistant condition, metabolite concentrations of primary cultured cells of group B (P/+) and group C (R/+) were compared under sunitinib exposure. Twenty-five metabolites showed stable and repeatable measurements. In the quantitative measurement system, 11 metabolites were significantly increased in group C (R/+), and metabolite concentrations were increased >2-fold compared with those in group B (P/+) (*p* < 0.05 for all) (Supplementary Figure S1). Among these increased metabolites, we identified and focused 8 metabolites that glutamine, glutamic acid, and 2-oxoglutaric acid were involved in glutamine uptake into the TCA cycle; fructose 6-phosohate, d-sedoheptulose 7-phosphate, and glucose 1-phosphate were involved in glycolysis and its intermediate metabolites; and glutathione and myoinositol have antioxidant effects (Figure 4A). The concentration levels of lactic acid and 2-hydroxyglutaric acid (2-HG) tended to increase by more than 1.5-fold in in group C (R/+) compared with those in group B (P/+), although there was no statistically significant difference. Then, these 8 metabolites that were significantly higher in group C (R/+) were analyzed between the three groups (groups A–C, Figure 4B). Regarding the metabolic pathway of glutamine into TCA cycle, glutamine and 2-oxoglutaric acid levels in group C (R/+) were significantly higher than those in group B (P/+) (*p* < 0.01) and comparable to those in group A (P/−). Conversely, glutamic acid levels in group A (P/−) were significantly higher than those in groups B (P/+) and C (R/+) (*p* < 0.01). Concerning the glycolysis pathway and its intermediate metabolites, glucose 1-phosphate and fructose 6-phosphate levels in group C (R/+) were higher than those in groups A (P/−) and B (P/+) (*p* < 0.01). d-sedoheptulose 7-phosphate levels in group C (R/+) were higher than those in group B (P/+) (*p* < 0.01). Regarding the pathway containing glutathione and myoinositol, which are antioxidant metabolites, glutathione and myoinositol levels in group A (P/−) were the highest (*p* < 0.01). Glutathione levels in groups B (P/+) and C (R/+) were not significantly different. Moreover, myoinositol levels in group C (R/+) were increased compared with those in group B (P/+) (*p* < 0.05).

### 2.4. SLC1A5 and LAT1 Expression Related to Glutamine Uptake in Sunitinib-Resistant Cells

**Figure 5:**
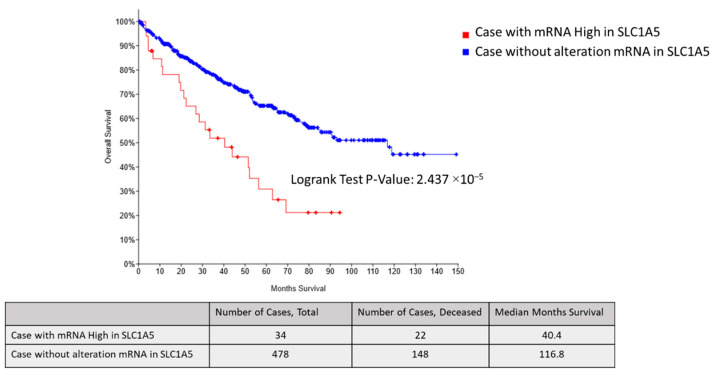
Kaplan–Meier survival plots for SLC1A5 mRNA high groups in a TCGA ccRCC cohort. Patients with high SLC1A5 mRNA expression (n = 34, red line) had significantly poorer overall survival compared with patients without altered SLC1A5 mRNA expression (n = 478, blue line) in ccRCC (*p* = 0.00002).

We focused on the glutamine pathway with energy metabolism and antioxidant activity involved in glutathione production. Furthermore, we analyzed the expression of SLC1A5, a transporter that uptakes glutamine into cells, using bioinformatics data. Survival curves data from the TCGA database indicated that patients with high SLC1A5 mRNA expression (n = 34) had significantly poorer overall survival compared with patients without altered SLC1A5 mRNA expression in ccRCC (*p* = 0.00002) (Figure 5).

**Figure 6:**
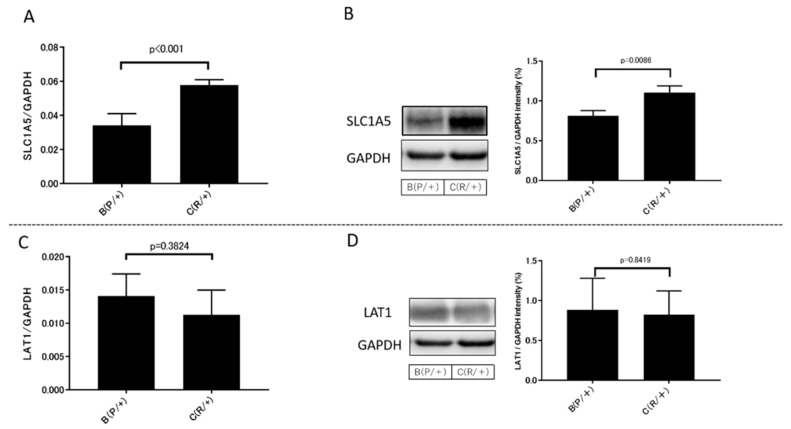
SLC1A5 is overexpressed in sunitinib-resistant cells: (**A**) qRT-PCR analysis demonstrated that SLC1A5 expression was significantly increased in group C (R/+) compared with that in group B (P/+) (*p* < 0.001); (**B**) Western blot analysis indicated that SLC1A5 protein expression was significantly increased in group C (R/+) compared with that in group B (P/+) (*p* = 0.0086); (**C**) LAT1 expression was not significantly different between the two groups (*p* = 0.3824); (**D**) LAT1 protein expression was not significantly different between the two groups (*p* = 0.8419).In qRT-PCR and western blot analysis, at least three independent experiments were performed. SLC1A5 and LAT1 expression levels were normalized to GAPDH expression in each sample. Data were analyzed for statistical significance by the Mann–Whitney U test.

In primary cells, the expression of glutamine transporters SLC1A5 and LAT1, a transporter that excretes intracellular glutamine, in groups B (P/+) and C (R/+) were compared by qRT-PCR and western blot analysis. SLC1A5 expression was significantly increased in group C (R/+) compared with that in group B (P/+) by qRT-PCR (*p* < 0.001) (Figure 6A). Similarly, SLC1A5 protein expression was significantly increased in group C (R/+) compared with that in group B (P/+) by western blot analysis (*p* = 0.0086) (Figure 6B). However, LAT1 expression was not significantly different between the two groups (Figure 6C,D).

## 3. Discussion

Metabolomics enables a comprehensive evaluation of cancer cell activity by analysis of intracellular metabolite products [25]. We comprehensively analyzed RCC for characteristic metabolites identified from our previous G-Met study [16]. In the present study, we precisely quantified and compared these intracellular metabolite concentrations between sunitinib-sensitive and sunitinib-resistant cell lines using our LC-MS/MS system. The measurement system using an internal stand substance enabled quantitative measurement even under different cell conditions.

In clinical practice, sunitinib remains a crucial TKI in sequential therapy for advanced RCC even in the era of therapy using immune checkpoint inhibitors. Despite initial efficacy, TKIs are only moderately effective in patients who rapidly develop drug resistance [26,27]. Therefore, patients with TKI-resistant RCC have a poor prognosis. To our knowledge, metabolites involved in mechanisms of sunitinib resistance has remained unclear. Thus, identification of significant metabolites could be clinically important for development of new therapeutic targets against sunitinib-resistant RCC to improve the prognosis of patients with such disease.

Signal transduction has been reported as a major factor that regulates metabolite production in cancer cells. Akt phosphorylation promotes glucose uptake through the glucose transporter GLUT1 and is associated with activation of hexokinase-2 and phosphofructokinase-2, which regulate glycolysis [28,29]. Downregulating mitochondrial function also affects glycolysis [19,20]. MET and AXL receptor tyrosine kinase (AXL) molecules, which are highly expressed in tumor cells, have been suggested as therapeutic targets for sunitinib-resistant RCC [21]. MET is a tyrosine-kinase receptor that promotes gene mutation and gene amplification activities, resulting in increased abnormal proteins [30,31]. High MET expression could be associated with poor prognosis in renal cancer, and an in vivo study showed MET phosphorylation enhanced by chronic sunitinib exposure could lead to enhanced migration of affected cells by activating signaling pathways, causing epithelial-mesenchymal transition [32,33].

Sunitinib targets the vascular endothelial growth factor (VEGF) signaling pathway, and once sunitinib resistance is established, the resulting compensatory increase of covered blood vessels and upregulation of alternative VEGF pathways could cause vascular endothelial cells to develop resistance to VEGF-targeted drugs [34]. Hatakeyama et al. noted enhanced glycolysis and TCA cycle and pentose phosphate pathway activity in an in vitro study of sunitinib-resistant 786-O cells [24]. However, potential effects on vascular endothelial cells were not evaluated in their study. Therefore, we established sunitinib-resistant RCC cells through in vitro and in vivo culture to explore the effects of sunitinib on vascular endothelium to identify alterations between intracellular metabolites.

**Figure 7:**
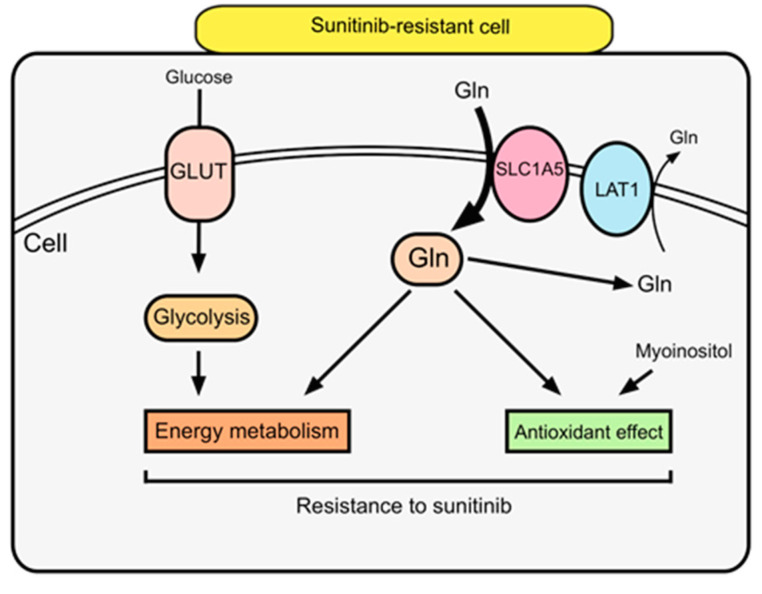
Metabolomic approach reveals mechanism of sunitinib resistance in RCC. In sunitinib-resistant cells, glutamine uptake into the cell through SLC1A5 contributes to both energy metabolism with increasing glutamine uptake and antioxidant effects related to sunitinib resistance.

Energy metabolism with glutamine uptake and glycolysis upregulation, as well as antioxidant activity, was indicated to contribute to sunitinib resistance in RCC in this study. We focused on the glutamine pathway because these metabolites correlated with both energy production and antioxidant effect. Thus, glutamine uptake into the TCA cycle is increased in sunitinib-resistant cells and may be the key for overcoming sunitinib resistance. Glutamine is involved in energy production via nucleic acid, protein, and lipid synthesis. Glutamine is converted by glutaminase to glutamic acid and serves as a precursor of glutathione, which has an antioxidant effect [35,36]. Regarding intracellular metabolism in sunitinib-resistant cells, glutamic acid is probably consumed in two metabolic processes mechanism with energy production via 2-oxoglutaric acid and antioxidant via glutathione [19,20,37]. These processes contribute to cell proliferation and bring a profit to cancer cells by inhibiting apoptosis due to oxidative stress. According to comparison of metabolites between group A (P/−) and group C (R/+), our results revealed that metabolism of sunitinib-resistant cells under sunitinib were similar to those of renal cancer cells without sunitinib. Glutamine, a non-essential amino acid, is taken up by cells via solute carrier (SLC)-type transporters [38]. One such transporter, SLC1A5, is highly expressed in cancer cells [35]. Furthermore, increased SLC1A5 expression was shown to be a poor prognostic factor for overall survival in patients with advanced ccRCC in the TCGA cohort (Figure 5). In the present study, SLC1A5 was significantly increased in sunitinib-resistant cells, whereas no differences in LAT1 expression was observed between sunitinib-sensitive and sunitinib-resistant cell lines. LAT1 is a transporter that excretes intracellular glutamine or neutral essential amino acids [39,40]. The functional coupling of SLC1A5 and LAT1 was cited in past reports [41,42]. Furthermore, SLC1A5 and LAT1 transporters were correlated with c-Myc signal which regulates the transcription of the *Slc1A5* gene [41,43]. Our results revealed that intracellular glutamine concentration was generated by high SLC1A5 expression. Proposed metabolic pathways implicated in sunitinib resistance are depicted in Figure 7.

Regulation of glutamine uptake related to SLC1A5 or glutaminase could also serve as molecular targets for the treatment of sunitinib-resistant RCC. V-9302, a competitive small molecule antagonist of SLC1A5, was shown to attenuate cancer cell growth and induce cancer cell apoptosis due to increased oxidative stress [42]. Blocking glutaminase to halt the conversion of glutamine to glutamic acid facilitates cancer cell apoptosis by reducing glutathione levels (a glutamic acid derivative), leading to reactive oxygen species generation [44]. Therefore, we consider that glutamine metabolism regulation may help to overcome sunitinib resistance in RCC.

There are several limitations to this study. First, the LC-MS measurement system using our institutional settings has not been externally validated. Second, the precise mechanism of high SLC1A5 expression in sunitinib-resistant cells was not evaluated. Third, we did not indicate the relationship with signal transduction and gene alternation in sunitinib resistance. Finally, sunitinib resistance was evaluated in only a few cell lines and other TKIs were not considered in this study.

All the cited limitations, we succeeded in demonstrating that energy metabolism with glutamine uptake and glycolysis upregulation, as well as antioxidant activity, could contribute to sunitinib resistance in RCC. Our results revealed activated glutamine transporter is found to be involved in sunitinib resistance. Further investigation of the regulation of these metabolites and glutamine transporters could lead to a new therapeutic strategy for advanced RCC patients with TKI treatment failure.

## 4. Materials and Methods

### 4.1. Ethics Statement

This study was conducted according to the principles expressed in the Declaration of Helsinki and animal experiments were approved by the Animal Care and Experimentation Committee of the Tohoku University Graduate School of Medicine (2018MdA-146).

### 4.2. Cell Lines and Culture

Human renal cell carcinoma cell lines (786-O, ACHN, and Caki-1) were purchased from American Type Culture Collection (ATCC; Manassas, VA, USA) and maintained in culture media. 786-O cells were maintained in an incubator at 37 °C and 5% CO_2_ in RPMI 1640 medium (Roswell Park Memorial Institute, Gibco/Life Technologies, Carlsbad, CA, USA) supplemented with 10% fetal bovine serum (FBS). ACHN and Caki-1 cells were maintained in Dulbecco’s modified Eagle’s medium (DMEM; Gibco/Life Technologies) supplemented with 10% FBS and 1% penicillin-streptomycin Mixed Solution (Nacalai Tesque, Kyoto, Japan). Experiments with the majority of cell lines were conducted within 3–6 months and 10 passages of purchase from ATCC, and each cell line was seeded at 2 × 10^6^ cells/dish for subsequent analysis. Sunitinib-resistant RCC cells were generated by growing parental 786-O (786-P) cells serially treated with increasing concentrations of sunitinib (S1042, Selleck, Houston, TX, USA) up to 10 µM. After continuous culture in complete medium supplemented with 10 µM sunitinib for >20 passages, these cells were used as sunitinib-resistant RCC cells (786-R) and maintained in medium containing 10 µM sunitinib [22,23,24].

### 4.3. WST Assay

WST assay was performed to measure sunitinib cytotoxicity. 786-O, ACHN, and Caki-1 cells were seeded onto a 96-well plate (Gibco, Thermo Fisher Scientific, Waltham, MA, USA) at a density of 5.0 × 10^3^ cells/well. After 24-h incubation, the culture medium was removed, and cells were treated with fresh medium containing different concentrations of sunitinib dissolved in dimethyl sulfoxide (DMSO, Sigma-Aldrich, St. Louis, MO, USA) for 48 h. The number of cells was analyzed by WST assay using Cell Counting Kit-8 (Dojindo, Kumamoto, Japan). The absorbance was measured at a wavelength of 450 nm using a microplate spectrophotometer (Multiskan GO, Thermo Fisher Scientific, Waltham, MA, USA). All experiments were repeated three times. The 50% cell growth inhibitory concentration (IC_50_) for each compound was evaluated.

### 4.4. Cell Proliferation Assay

To compare in vitro proliferation of 786-O sublines (786-P and 786-R), 1 × 10^4^ cells of each cell line were seeded with 5 µM sunitinib in each well of 24-well plates. The number of cells in each cell line was assessed daily in triplicate using Cell Counting Kit-8 using an Infinite 200 fluorescence plate reader (Tecan, Männedorf, Switzerland). All experiments were repeated three times.

### 4.5. Establishment of Sunitinib-Resistant Mouse Model

Female BALB/c-nu/nu mice (4–6-week-old) were purchased from CLEA Japan (Tokyo, Japan). Mice were reared 1 week after carrying in the breeding room as an environmental adaptation period. 786-P or 786-R cells were trypsinized and 2 × 10^6^ cells were subcutaneously injected with 100 mL Matrigel (Corning, Corning, NY, USA). Tumor diameter was measured twice a week with a digital caliper, and tumor volume was calculated using the modified ellipsoid formula 1/2 (length × width^2^) after transplantation. Regarding the validity of the sunitinib dose, a dose of 20–80 mg/kg/day affected for tumor reduction in the xenograft RCC mouse model, and a dose of 20–25 mg/kg/day created a sunitinib-resistant mouse model in previous reports [4,21,45]. Therefore, sunitinib (25 mg/kg/day) was orally administered in the study. Sunitinib was adjusted with 4% DMSO + 30% PEG300 + ddH_2_O and orally administered using sonde. Mice were divided into the following three experimental groups (n = 5/group). Group A (P/−) was subcutaneously transplanted with 786-P and did not receive sunitinib. Group B (P/+) was subcutaneously transplanted with 786-P and received sunitinib treatment. Group C (R/+) was subcutaneously transplanted with 786-R and received sunitinib treatment. From the time the tumor volume exceeded 62.5 mm^3^ (tumor diameter visualized to be ≥5 mm), each group was treated as follows. Group A (P/−) was observed for 6 weeks. Groups B (P/+) and C (R/+) were observed for 2 weeks and subsequently administered sunitinib for 4 weeks. Thereafter, animals were euthanized, subcutaneous tumors were removed, and primary cell culture was started using the trypsin method. Subsequent experiments were performed using each cell line within 2 passages without sunitinib after the primary cell culture.

### 4.6. Wound-Healing Assay

Cells were seeded in wells of 6-well plates in normal cell-growth medium and grown until confluent. Then, a 1-mL pipette tip was used to make a straight scratch, simulating a wound. The medium was changed to RPMI 1640 with 5 µM sunitinib. After 24- or 48-h incubation, the area occupied by cells that had migrated into the scratch area was measured using ImageJ software (National Institutes of Health, Bethesda, MD, USA). The ratio of the migration area to the scratch area was graphed. The experiment was carried out in triplicate and repeated three times.

### 4.7. Two-Chamber Assay

Cell migration was assessed using a two-chamber assay with a Transwell 3422 (Corning, Corning, NY, USA) membrane with cell-culture insert (8-μm pore size) in 24-well plates. Approximately 5 × 10^4^ cells were plated in each insert in serum-free medium with 5 µM sunitinib. The bottom well contained medium supplemented with 10% FBS and fibronectin (Corning, Corning, NY, USA). After 48 h, the bottom of the insert was stained with 1% crystal violet/D-PBS for 30 min, and cells that invaded through the membrane to the lower surface were counted. The experiment was carried out in triplicate and repeated three times.

### 4.8. Phosphorylation Stimulation and Antibodies

Cells were subjected to starvation treatment (cultured in RPMI 1640 medium without addition of FBS) for 24 h, and then phosphorylation stimulation was performed. Hepatocyte growth factor (HGF) was purchased from PeproTech (Shanghai, China) as a stimulus for MET phosphorylation. Phosphorylation stimulation with HGF was performed at 40 ng/mL for 10 min. For Akt phosphorylation, cells were plated in medium supplemented with 10% FBS for 10 min. Antibodies anti-Akt (pan, rabbit IgG, cat no. #4691), phospho-Akt (rabbit IgG, Ser 473, #4060), anti-MET (rabbit IgG, #8198), phospho-MET (rabbit IgG, Tyr 1234/1235, #3077), anti-SLC1A5 (#4692), and anti-LAT1 (#5347) were purchased from Cell Signaling Technology (Danvers, MA, USA), and GAPDH (sc-32233) was purchased from Santa Cruz Biotechnology (Santa Cruz, CA, USA).

### 4.9. Western Blot Analysis

Cells were lysed in radioimmunoprecipitation lysis buffer (Santa Cruz Biotechnology, Dallas, TX, USA) supplemented with phosphatase inhibitor cocktail (Kaygen, Irvine, CA, USA). Protein amount in lysates was quantified by the Pierce BCA protein assay kit (Thermo Fisher Scientific, Waltham, MA, USA). Then, 20 µg of protein per sample were dissolved at 95 °C in 4 × Laemmli sample buffer (Bio-Rad Laboratories, Hercules, CA, USA) and β-mercaptoethanol (Wako Pure Chemical Industries, Ltd., Osaka, Japan). Separated proteins were then transferred to polyvinylidene difluoride (PVDF) membranes (Hybond-P PVDF, Amersham Biosciences, Little Chalfont, UK) using the TRANS-Blot^®^ Turbo™ System (Bio-Rad Laboratories, Hercules, CA, USA). PVDF membranes were blocked with 1% bovine serum albumin (Wako Pure Chemical Industries, Ltd., Osaka, Japan) and incubated at 4 °C with shaking for at least 12 h to react with the primary antibody. Membranes were then incubated with secondary antibody [goat anti-mouse IgG (H + L), horseradish peroxidase conjugate (Life Technologies, Inc., Eugene, OR, USA) or goat anti-rabbit IgG (H + L), horseradish peroxidase conjugate (Life Technologies, Inc., Carlsbad, CA, USA)] at room temperature with shaking for 60 min. Immunoreactive bands were developed using Clarity™ Western ECL Substrate (Bio-Rad Laboratories, Hercules, CA, USA), detected using the ChemiDoc™ MP Imaging System (Bio-Rad Laboratories, Hercules, CA, USA), and quantified using Image Lab software version 4.1 (Bio-Rad Laboratories, Hercules, CA, USA). In a series of western blot analyses, the same experiment was performed at least three times.

### 4.10. Chemicals and Reagents for LC-MS/MS Analysis

Forty-seven metabolites, which were increased in our ccRCC tissue study, were evaluated in this study [16]. Standard substances for calibration curve and isotope-labeled internal standards for quantitative measurement are listed in Supplementary Data S1.

### 4.11. LC-MS/MS Conditions

All LC-MS/MS analyses were performed using the LCMS-8050 triple quadrupole mass spectrometer coupled with the Nexera X2 UHPLC system (Shimadzu, Kyoto, Japan) and Lab Solutions software (Shimadzu, Kyoto, Japan). To analyze metabolites, analytes were separated into four groups and analyzed with each optimized method for improvement of the measurement sensitivity with each metabolite. The LC method used for each compound and optimized MS/MS conditions are summarized in Supplementary Table S1. For all four methods, column oven temperature was set at 40 °C and electrospray ionization mode was chosen as the ion source probe. Ion source probe conditions were as follows: probe voltage, 4000 V; desolvation line temperature, 100 °C; block heater temperature, 150 °C; interface temperature, 400 °C; nebulizing gas flow, 2 L/min; drying gas flow, 3 L/min; and heating gas flow, 17 L/min. Column and mobile phase were selected for metabolites with high sensitivity, and calibration and internal standards for each group are listed in Supplementary Data S2 and Supplementary Table S2.

### 4.12. Sample Preparation for LC-MS/MS

For intracellular metabolite concentration measurement, cells were divided into four groups. In groups 1 and 2, cells were seeded at 5 × 10^4^ cells/well in 6-well plates and cultured at 37 °C with 5% CO_2_. Groups 3 and 4 were seeded at 2 × 10^5^ cells/dish in a 10-cm dish and cultured at 37% with 5% CO_2_. After 24 h, culture supernatant was removed and medium containing DMSO or 5 µM sunitinib was added. After culturing for 48 h, cells were washed twice with D-PBS and recovered with a scraper. Cell pellets were stored at −80 °C until measurement.

In groups 1 and 2, 100 µL internal standard substance (IS) mix and 100 µL of 50% acetonitrile were added to cell pellets, then pellets were vortexed and disrupted using an ultrasonic vibrator. Samples were then centrifuged for 5 min at 15,000× *g* and 4 °C and 100 µL of supernatant was transferred to a 1.5-mL PP tube (Lot 509-GRD-Q; Thermo Fisher Scientific, Waltham, MA, USA). Supernatants were then dried using a centrifugal evaporator for 1 h and redissolved in 50 µL water for group 1 samples or 50 µL 75% acetonitrile for group 2 samples. For group 3, 25 µL IS, 200 µL water, and 1 mL acetonitrile were added to cell pellets and vortexed. Cell disruption was subsequently performed using an ultrasonic vibrator. Samples were then centrifuged for 5 min at 15,000× *g* at 4 °C, and supernatants were transferred to a 1.5-mL PP tube. Samples were then dried using a centrifugal evaporator and reconstituted in 20 µL water. For group 4, 140 µL 50% acetonitrile, 10 µL IS, 175 µL water, and 175 µL acetonitrile were added to cell pellets. Samples were vortexed and cell disruption was performed using an ultrasonic vibrator. Samples were then centrifuged for 5 min at 15,000× *g* and 4 °C and supernatants were transferred to a 1.5-mL PP tube. Approximately 50 µL supernatant were used for measurement. The obtained intracellular metabolite concentration was counted for each cell, and the concentration per 10,000 cells was calculated. Cells were cultured in triplicate, and measurements were performed three times to calculate the mean metabolite concentration.

### 4.13. RNA Extraction and Quantitative RT-PCR

RNA was extracted from cell lysates using the acid guanidinium-phenol-chloroform method, and total RNA was reverse transcribed into cDNA using the iScript™ cDNA Synthesis Kit (Bio-Rad Laboratories, Hercules, CA, USA) following the manufacturer’s protocol [46]. Quantitative real-time PCR (qRT-PCR) was performed using the Dice Real Time System Thermal Cycler (TP900, Takara Bio Inc., Shiga, Japan) and SYBR Premix Ex Taq™ II (Takara, Shiga, Japan). Reactions were performed in 25-µL volume. The protocol consisted of 40 replication cycles. The following primers were used: solute carrier family 1 member 5 (SLC1A5), 5′-GACCGTACGGAGTCGAGAAG-3′ (forward) and 5′-GGGGGTTTCCTTCCTCAGTG-3′ (reverse) [47]; L-type amino acid transporter 1 (LAT1), 5′-GCCCATTGTCACCATCATC-3′ (forward) and 5′-GAGCCCACAAAGAAAAGC-3′ (reverse) [48]; human kidney-type glutaminase (KGA), 5′-CGAAGATTTGCTTTGTCAGCTATGG-3′ (forward) and 5′-CTCTGCAGCAGCTACATGGA-3′ (reverse) [49]; and GAPDH (internal control), 5′-TTCTTTTGCGTCGCCAGCCGA-3′ (forward) and 5′-GTGACCAGGCGCCCAATACGA-3′ (reverse).

### 4.14. Bioinformatics Analysis

Information for 512 patients with ccRCC (Pan-Cancer Atlas) in the Cancer Genome Atlas (TCGA) cohort database was used for analysis. Data utilized in this paper can be accessed via cBioPortal (www.cBioPortal).

### 4.15. Statistical Analysis

All data from both in vitro and in vivo analyses were analyzed for statistical significance by the Mann–Whitney U test. Differences in experimental groups were determined by one-way ANOVA followed by the post hoc Tukey’s test, as appropriate. All statistical analyses were performed using GraphPad Prism software (Version 7.0; GraphPad Software Inc., La Jolla, CA, USA). Statistical significance was considered to exist at *p* < 0.05.

## Data Availability

The data presented in this study are available in supplementary material.

## Supplementary Materials

The following are available online at https://www.mdpi.com/2218-1989/11/1/1/s1, Figure S1: Metabolite concentrations were compared between primary cultured cells of groups B (P/+) and C (R/+) under sunitinib exposure., Data S1: Standard substances for calibration curve and isotope-labeled internal standards for quantitative measurement., Table S1: MS/MS conditions of 4 simultaneous analytical methods., Data S2: Colum and mobile phase., Table S2: Setting of internal standards for each analyte.
