# Supplementary material for: Metabolomic Analysis to Elucidate Mechanisms of Sunitinib Resistance in Renal Cell Carcinoma
Source: Metabolites. 2020 Dec 22;11(1):1. doi: 10.3390/metabo11010001 (PMC7821950; doi:10.3390/metabo11010001)
Supplement: Supplementary file 1 [file metabolites-11-00001-s001.zip › Supplementary/Supplementary data 1.docx]

Supplementary data 1

2-ketoglutaric acid-^13^C_1_, fructose 6-phosphate-^13^C_6_, D-galactose-^2^H, myoinositol-^2^H_6_, N-hexanoylglycine-^2^H_11_, sn-glycero-3-phosphocholine-^2^H_9_ and *N*-hexanoylglycine were purchased from Toronto Research Chemicals (Toronto, Canada). Succinic acid, D-glucose 1-phosphate, D-fructose 6-phosphate, D-galactose and myoinositol were purchased from Nakalai Tesque (Kyoto, Japan). 2-hydroxyglutaric acid (2-HG), L-lactic acid, ophthalmic acid, *S*-lactoylglutathione, D-sedoheptulose 7-phosphate, phosphorylcholine, glycerophosphorylcholine, D-saccharic acid, phosphorylcholine-^2^H_9_ and gluthathione-[^13^C_2_, ^15^N] were purchased from Sigma-Aldrich (St. Louis, MO, USA). Glutathione (reduced form), glutathione (oxidized form), glutamine and glutamic acid　were purchased from FUJIFILM Wako Pure Chemical Industries (Osaka, Japan). 2-oxoglutaric acid and 3-methoxybenzenepropanoic acid were purchased from Tokyo Chemical Industry Co. (Tokyo, Japan) and Ark Pharma (Arlington Heights, IL, USA), respectively. Glutamine-^2^H_5_　and Glutamic acid-^2^H_5_ were Cambridge isotope laboratories　(Tewksbury, MA, USA). Lactate-^2^H_3_ was purchased from CDN-isotope (Montreal, Canada). 2-hydroxyglutaric acid-^2^H_3_ was purchased from Shinsei Chemical Company Ltd. (Osaka, Japan). Succinic acid-2,2,3,3-^2^H_4_　were purchased from Santa Cruz Biotechnology (Dallas, Texas, USA).

L-Tryptophan, anthranilic acid, picolinic acid and indole-3-acetic acid were purchased from Nakalai Tesque (Kyoto, Japan). L-Kynurenine, 3-hydroxyanthranilic acid, kynurenic acid, *N*-formylanthranilic acid and cinnabarinic acid were purchased from Sigma-Aldrich (St. Louis, MO, USA). Xanthurenic acid was purchased from MP Biomedicals (Santa Ana, CA, USA). *N*-Formylkynurenine, kynurenic acid-^2^H_5,_ 3-hydroxyanthranilic acid-^2^H_3,_ picolinic acid-^2^H_3,_ quinolinic acid-^2^H_3_ and xanthurenic acid-^2^H_4_ were purchased from Toronto Research Chemicals (Toronto, Canada). Tryptophan-^2^H_5_ and indole-3-acetic acid-^2^H_5_ were purchased from CDN-isotope (Montreal, Canada).

L-Carnitine was purchased fromWako (Osaka, Japan), and acetyl-L-carnitine hydrochloride was purchased from Enzo Life Science (Farmingdale, NY, USA). Propionyl-L-carnitine, butyryl-L-carnitine, lauroyl-L-carnitine hydrochloride, and stearoyl-L-carnitine were purchased from Larodan (Stockholm, Sweden). Hexanoyl-DL-carnitine, octanoyl-DL-carnitine, decanoyl-DL-carnitine, myristoyl-DLcarnitine and palmitoyl-DL-carnitine were purchased from Tocris Bioscience (Minneapolis, MN, USA). DL-[^2^H_9_] carnitine hydrochloride was purchased from Toronto Research Chemicals (Toronto, Canada), and hexanoyl-L-[^2^H_3_] carnitine and stearoyl-L-[^2^H_3_] carnitine were purchased from CDN isotopes (Quebec, Canada). Pivaloylcarnitine (C5) was synthesized from pivaloyl hydrochloride and L-carnitine according to the method reported by Todesco et al.

Ultrapure water for liquid chromatography tandem mass spectrometry (LC–MS/MS) was prepared with a PURELAB Ultra Genetic system (Organo, Tokyo, Japan). Acetonitrile, formic acid, methanol (high performance liquid chromatography grade) and ammonium formate were purchased from FUJIFILM WAKO Pure Chemical Co., Ltd. (Osaka, Japan).
