# Supplementary material for: Metabolomic Analysis to Elucidate Mechanisms of Sunitinib Resistance in Renal Cell Carcinoma
Source: Metabolites. 2020 Dec 22;11(1):1. doi: 10.3390/metabo11010001 (PMC7821950; doi:10.3390/metabo11010001)
Supplement: Supplementary file 1 [file metabolites-11-00001-s001.zip › Supplementary/Supplementary data 2.docx]

Supplementary data 2

Colum and mobile phase

For analysis of group 1, CAPCELL PAK ADME (2.1 mm × 50 mm, 2 μm, Osaka soda, Osaka) was used as an analytical column. A mixture of water/formic acid (100:0.1, v/v) and acetonitrile were used as mobile phase A and B at the 0.4 mL/min of flow rate. The gradient program was as follows: 0–2.5min, 1% B; 2.5–2.6min, 1–95% B; 2.6–4 min, 95% B; 4–4.1 min, 95–1% B; 4.1–5 min, 1% B. The injection volume was 3μL for analysis.

For analysis of group 2, CORTECS silica (2.1 mm × 100 mm, 1.6 μm, Waters Corp., Milford, MA) was used as a hydrophilic interaction chromatograph column. The flow rate was set at 0.4 mL/min. The mobile phase consisted of a mixture of 20 mmol/L ammonium formate/formic acid (100:0.1, v/v) adjusted to pH 3.5 (A) and acetonitrile (B). The gradient program was as follows: 0–7 min, 95–25% B; 7–7.1 min, 25–5% B; 7.1–8 min, 5% B; 8–8.1 min, 5–95% B; 8.1–15 min, 95% B. The injection volume for analysis was set at 3μL.

For analysis of group 3, CAPCELL PAK ADME (2.1 mm × 150 mm, 3 μm, Osaka soda, Osaka) was used for chromatographic separation of aromatic metabolites. The flow rate was set at 0.2 mL/min. The mobile phase consisted of a mixture of 0.2% acetylacetone/0.4% formic acid in water as A and a mixture of 0.2% acetylacetone/0.4% formic acid in acetonitrile as B. The gradient program was as follows: 0–3 min, 0% B; 3–3.2 min, 0–5% B; 3.2–7 min, 5% B; 7–13 min, 5–60% B; 13–13.1 min, 60–95% B; 13.1–18 min, 95% B; 18–18.1 min, 95–0% B; 18.1–30 min, 0% B. The injection volume for analysis was set at 10μL.

For analysis of group 4, Inertsil ODS-3 (2.1 mm i.d. × 150mm, 5 μm, GL Science, Tokyo) was used for carnitine pathway measurement. The mobile phases consisted of elute A (0.1% HFBA in water) and elute B (0.1% HFBA in acetonitrile). The flow rate was 0.2 ml/min. The gradient started at 10% B, and then proceeded as follows: 0–1 min, gradient to 10% B; 1–4 min, gradient to 90% B; 4–7 min, gradient to 90% B; 7–10 min, gradient to 90% The injection volume for analysis was set at 1μL^37^.

Preparation of calibration standards and internal standards

For preparation of working solution for group 1 and 2, 21 stock solutions of glutathione (reduced form), glutathione (oxidized form), L-lactic acid, 2-HG, glutamine, glutamic acid, phosphorylcholine, glycerophosphorylcholine, 2-oxoglutaric acid, acetyl coenzyme A, succinic acid, ophthalmic acid, D-glucose 1-phosphate, D-fructose 6-phosphate, D-galactose, S-lactoylglutathione, myoinositol, D-sedoheptulose 7-phosphate, 3-methoxybenzenepropanoic acid, D-saccharic acid, N-hexanoylglycine of each stock solution (around 10 mM) were mixed and diluted with acetonitrile/water (50:50, v/v)　to 100 nM, 300 nM, 1 µM, 3 µM, 10 µM, 30 µM and 100 µM. As internal standard (IS) solution mixture 1 (IS mix 1), 15 stock solutions of lactate-^2^H_3_, 2-HG-^2^H_3_, gluthathione-[^13^C_2_, ^15^N], L-glutamine-^2^H_5_, L-glutamic acid-^2^H_5_, phosphorylcholine-^2^H_9_, sn-glycero-3-phosphocholine-^2^H_9_, 2-Ketoglutaric acid-^13^C_1_, fructose 6-Phosphate-^13^C_6_, D-galactose-d, myoinositol-^2^H_6_ and *N*-hexanoylglycine-^2^H_11_ were mixed and diluted to with water to 20 µM.

For preparation of working solution for group 3, standard solutions were prepared at concentration of 100 mmol/l for *N*-formylkynurenine, cinnabarinic acid, L-tryptophan, L-kynurenine, xanthurenic acid, 3-hydroxykynurenine, 5-hydroxyanthranilic acid, picolinic acid, nicotinic acid, anthranilic acid, 3-hydroxyanthranilic acid, quinolinic acid, indole-3-acetic acid, kynurenic acid and

*N*-formylanthranilic acid were diluted in water to 100 nM, 300 nM, 1 µM, 3 µM, 10 µM, 30 µM and 100 µM. IS stock solutions were prepared for tryptophan-^2^H_5,_ kynurenic acid-^2^H_5,_ xanthurenic acid-^2^H_4,_ picolinic acid-^2^H_3,_ nicotinic acid-^2^H_4,_ 3-hydroxyanthranilic acid-^2^H_3,_ quinolinic acid-^2^H_3,_ indole-3-acetic acid-^2^H_5,_ anthranilic acid-^2^H_4,_ kynurenine-^2^H_4_ were mixed and diluted with water to a final concentration of 10 μM

For preparation of working solution for group 4, standard solutions were prepared at concentration of 1-4 mmol/l for carnitine, acetylcarnitine, propionylcarnitine butyrylcarnitine, pivaloylcarnitine, hexanoylcarnitine, octanoylcarnitine, decanoylcarnitine, lauroylcarnitine, myristoylcarnitine palmitoylcarnitine and stearoylcarnitine in water/acetonitrile 1:1 (v/v) to 100 nM, 300 nM, 1 µM, 3 µM, 10 µM, 30 µM and 100 µM. IS stock solutions were prepared at concentrations of 500 μmol/l for DL-[^2^H_9_] carnitine hydrochloride and 1 mmol/l for Hexanoyl-L-[^2^H_3_] carnitine and Stearoyl-L-[^2^H_3_]carnitine, all in water. Before use, the stocks were mixed and diluted with water to a final concentration of 10 μM.
