# Supplementary material for: Metabolomic Analysis to Elucidate Mechanisms of Sunitinib Resistance in Renal Cell Carcinoma
Source: Metabolites. 2020 Dec 22;11(1):1. doi: 10.3390/metabo11010001 (PMC7821950; doi:10.3390/metabo11010001)
Supplement: Supplementary file 1 [file metabolites-11-00001-s001.zip › Supplementary/Supplementary table 1.docx]

Supplementary table 1

MS/MS conditions of 4 simultaneous analytical methods

1. Group 1–analyzed with ADME column

| Compound | Q1  (*m/z*) | Q3  (*m/z*) | Q1 PB  (V) | CE  (V) | Q3 PB  (V) | Polarity |
| --- | --- | --- | --- | --- | --- | --- |
| GSSG | 613.3 | 355.3 | 26 | 24 | 25 | Positive |
| GSH | 308.0 | 162.0 | 11 | 17 | 28 | Positive |
| 2-Oxoglutaric acid | 145.1 | 101.1 | -30 | -6 | -16 | Negative |
| L-Lactate | 89.2 | 43.0 | -10 | -13 | -16 | Negative |
| (*R*)-2-hydroxyglutaric acid | 147.0 | 128.9 | -14 | -14 | -22 | Negative |
| Succinic acid | 117.1 | 73.2 | -13 | -14 | -11 | Negative |
| Ophthalmic acid | 287.9 | 195.3 | -14 | -16 | -13 | Negative |
| D-Saccharic acid | 209.0 | 165.2 | -15 | -16 | -15 | Negative |
| GSH-[^13^C_2_,^15^N] | 311.1 | 165.1 | 13 | 17 | 28 | Positive |
| 2-Oxoglutaric acid-^13^C | 145.9 | 102.2 | -15 | -16 | -15 | Negative |
| L-Lactic acid-^2^H_3_ | 92.1 | 92.1 | -17 | -11 | -26 | Negative |
| 2-hydroxyglutaric acid-^2^H_3_ | 150.0 | 131.2 | -17 | -15 | -12 | Negative |
| Succinic acid-^2^H_4_ | 121.2 | 77.2 | -15 | -11 | -15 | Negative |

1. Group 2–analyzed with HILIC column

| Compound | Q1  (*m/z*) | Q3  (*m/z*) | Q1 PB  (V) | CE  (V) | Q3 PB  (V) | Polarity |
| --- | --- | --- | --- | --- | --- | --- |
| (*S*)-Lactoylglutathione | 379.9 | 233.1 | 15 | 20 | 15 | Positive |
| L-Glutamic acid | 148.1 | 84.2 | 14 | 17 | 21 | Positive |
| L-Glutamine | 147.1 | 84.0 | 20 | 18 | 15 | Positive |
| Phosphorylcholine | 183.9 | 86.1 | 21 | 17 | 30 | Positive |
| Glycerophosphorylcholine | 257.9 | 104.0 | 20 | 16 | 20 | Positive |
| *N*-Hexanoylglycine | 172.4 | 74.1 | 11 | 13 | 26 | Positive |
| D-Fructose-6-phosphate | 258.8 | 79.0 | -28 | -54 | -30 | Negative |
| α-D-Glucose-1-phosphate | 258.8 | 79.0 | -12 | -39 | -17 | Negative |
| D-Sedoheptulose-7-phosphate | 289.2 | 79.0 | -10 | -22 | -16 | Negative |
| D-Galactose | 225.0 | 179.3 | -15 | -24 | -15 | Negative |
| Myoinositol | 225.0 | 45.1 | -15 | -25 | -15 | Negative |
| 3-Methoxybenzenepropanoic acid | 179.0 | 179.3 | -15 | -20 | -15 | Negative |
| L-Glutamic acid-^2^H_5_ | 153.3 | 88.1 | 12 | 16 | 15 | Positive |
| L-Glutamine-^2^H_5_ | 151.9 | 135.2 | 13 | 14 | 14 | Positive |
| Phosphorylcholine-^2^H_9_ | 192.9 | 95.2 | 21 | 18 | 22 | Positive |
| Glycerophosphorylcholine-^2^H_9_ | 266.9 | 113.2 | 12 | 16 | 22 | Positive |
| *N*-Hexanoylglycine-^2^H_11_ | 183.5 | 75.3 | 12 | 15 | 14 | Positive |
| L-Lactic acid-^2^H_3_ | 92.1 | 92.1 | -17 | -11 | -26 | Negative |
| D-Fructose-6-phosphate-^13^C_6_ | 264.9 | 79.1 | -13 | -38 | -11 | Negative |
| D-Galactose-^2^H | 226.0 | 136.1 | -15 | -16 | -15 | Negative |
| Myoinositol-^2^H_6_ | 230.9 | 45.2 | -15 | -12 | -15 | Negative |

1. Group 3–analyzed with ADME column

| Compound | Q1  (*m/z*) | Q3  (*m/z*) | Q1 PB  (V) | CE  (V) | Q3 PB  (V) | Polarity |
| --- | --- | --- | --- | --- | --- | --- |
| *N*-Formylkynurenine | 237.0 | 146.1 | -12 | -22 | -15 | Positive |
| Cinnabarinic acid | 301.0 | 237.0 | -22 | -29 | -25 | Positive |
| L-Tryptophan | 205.1 | 188.1 | -16 | -11 | -20 | Positive |
| L-Kynurenine | 209.1 | 146.1 | -16 | -18 | -15 | Positive |
| Xanthurenic acid | 206.0 | 178.1 | -23 | -18 | -30 | Positive |
| 3-Hydroxykynurenine | 225.1 | 162.1 | -17 | -21 | -16 | Positive |
| 5-Hydroxyanthranilic acid | 154.0 | 108.1 | -12 | -21 | -21 | Positive |
| Picolinic acid | 124.0 | 78.0 | -13 | -19 | -14 | Positive |
| Nicotinic acid | 124.0 | 80.1 | -20 | -20 | -20 | Positive |
| Anthranilic acid | 138.0 | 120.0 | -15 | -14 | -21 | Positive |
| 3-Hydroxyanthranilic acid | 154.0 | 108.1 | -18 | -20 | -22 | Positive |
| Quinolinic acid | 168.0 | 124.1 | -13 | -14 | -13 | Positive |
| Indole-3-acetic acid | 176.1 | 77.1 | -13 | -42 | -30 | Positive |
| Kynurenic acid | 190.0 | 144.1 | -10 | -17 | -25 | Positive |
| *N*-Formylanthranilic acid | 164.1 | 120.2 | 10 | 15 | 11 | Negative |
| Tryptophan-^2^H_5_ | 210.1 | 150.1 | -11 | -19 | -30 | Positive |
| Kynurenic acid-^2^H_5_ | 195.1 | 167.1 | -21 | -23 | -28 | Positive |
| Xanthurenic acid-^2^H_4_ | 210.1 | 182.1 | -24 | -17 | -19 | Positive |
| Picolinic acid-^2^H_3_ | 127.1 | 81.1 | -10 | -20 | -15 | Positive |
| Nicotinic acid-^2^H_4_ | 128.0 | 84.1 | -14 | -22 | -15 | Positive |
| 3-Hydroxyanthranilic acid-^2^H_3_ | 157.0 | 111.1 | -17 | -22 | -20 | Positive |
| Quinolinic acid-^2^H_3_ | 171.0 | 127.1 | -13 | -11 | -23 | Positive |
| Indole-3-acetic acid-^2^H_5_ | 181.1 | 106.1 | -14 | -31 | -19 | Positive |
| Anthranilic acid-^2^H_4_ | 142.1 | 124.2 | -14 | -13 | -23 | Positive |
| Kynurenine-^2^H_4_ | 213.2 | 150.2 | -11 | -17 | -15 | Positive |

1. Group 4–analyzed with Inertsil ODS-3

| Compound | Q1  (*m/z*) | Q3  (*m/z*) | Q1 PB  (V) | CE  (V) | Q3 PB  (V) | Polarity |
| --- | --- | --- | --- | --- | --- | --- |
| Carnitine | 162.1 | 84.8 | -20 | -30 | -20 | Positive |
| Acetylcarnitine | 204.2 | 84.8 | -20 | -25 | -20 | Positive |
| Propionylcarnitine | 218.2 | 84.8 | -20 | -30 | -20 | Positive |
| Butyrylcarnitine | 232.1 | 84.8 | -20 | -30 | -20 | Positive |
| Pivaloylcarnitine | 246.1 | 84.8 | -20 | -30 | -20 | Positive |
| Hexanoylcarnitine | 260.3 | 84.8 | -20 | -35 | -20 | Positive |
| Octanoylcarnitine | 288.2 | 84.8 | -20 | -30 | -20 | Positive |
| Decanoylcarnitine | 316.3 | 84.8 | -20 | -35 | -20 | Positive |
| Lauroylcarnitine | 344.1 | 84.8 | -20 | -35 | -20 | Positive |
| Myristoylcarnitine | 372.5 | 84.8 | -20 | -35 | -20 | Positive |
| Palmitoylcarnitine | 400.3 | 84.8 | -20 | -40 | -20 | Positive |
| Stearoylcarnitine | 428.1 | 84.8 | -20 | -40 | -20 | Positive |
| DL-[^2^H_9_] carnitine hydrochloride | 171.1 | 84.8 | -20 | -30 | -20 | Positive |
| Hexanoyl-L-[^2^H_3_] carnitine | 263.3 | 84.8 | -20 | -35 | -20 | Positive |
| Stearoyl-L-[^2^H_3_] carnitine | 431.1 | 84.8 | -20 | -40 | -20 | Positive |

CE, collision energy; PB, pre bias.

GSSG, glutathione oxidized form; GSH, glutathione reduced form.
