# Supplementary material for: Metabolomic Analysis to Elucidate Mechanisms of Sunitinib Resistance in Renal Cell Carcinoma
Source: Metabolites. 2020 Dec 22;11(1):1. doi: 10.3390/metabo11010001 (PMC7821950; doi:10.3390/metabo11010001)
Supplement: Supplementary file 1 [file metabolites-11-00001-s001.zip › Supplementary/Supplementary table 2.docx]

Supplementary table 2 Setting of internal standards for each analyte

1. Group 1–analyzed with ADME column

| Number | Compound |  |
| --- | --- | --- |
| Analyte 1 | GSSG | IS 1 |
| Analyte 2 | GSH | IS 1 |
| Analyte 3 | 2-Oxoglutaric acid | IS 2 |
| Analyte 4 | L-Lactic acid | IS 3 |
| Analyte 5 | (*R*)-2-hydroxyglutaric acid | IS 4 |
| Analyte 6 | Succinic acid | IS 5 |
| Analyte 7 | Ophthalmic acid | IS 5 |
| Analyte 8 | D-Saccharic acid | IS 5 |
| IS 1 | GSH-[^13^C_2_,^15^N] | - |
| IS 2 | 2-Oxoglutaric acid-^13^C | - |
| IS 3 | L-Lactic acid-^2^H_3_ | - |
| IS 4 | 2-hydroxyglutaric acid-^2^H_3_ | - |
| IS 5 | Succinic acid-^2^H_4_ | - |

1. Group 2–analyzed with HILIC column

| Number | Compound |  |
| --- | --- | --- |
| Analyte 1 | L-Glutamic acid | IS 1 |
| Analyte 2 | L-Glutamine | IS 2 |
| Analyte 3 | (*S*)-Lactoylglutathione | IS 2 |
| Analyte 4 | Phosphorylcholine | IS 3 |
| Analyte 5 | Glycerophosphorylcholine | IS 4 |
| Analyte 6 | *N*-Hexanoylglycine | IS 5 |
| Analyte 7 | 3-Methoxybenzenepropanoic acid | IS 6 |
| Analyte 8 | D-Fructose-6-phosphate | IS 7 |
| Analyte 9 | α-D-Glucose-1-phosphate | IS 7 |
| Analyte 10 | D-Sedoheptulose-7-phosphate | IS 7 |
| Analyte 11 | D-Galactose | IS 8 |
| Analyte 12 | Myoinositol | IS 9 |
| IS 1 | L-Glutamic acid-^2^H_5_ | - |
| IS 2 | L-Glutamine-^2^H_5_ | - |
| IS 3 | Phosphorylcholine-^2^H_9_ | - |
| IS 4 | Glycerophosphorylcholine-^2^H_9_ | - |
| IS 5 | *N*-Hexanoylglycine-^2^H_11_ | *-* |
| IS 6 | L-Lactic acid-^2^H_3_ | - |
| IS 7 | D-Fructose-6-phosphate-^13^C_6_ | - |
| IS 8 | D-Galactose-^2^H | - |
| IS 9 | Myoinositol-^2^H_6_ | - |

1. Group 3–analyzed with ADME column

| Number | Compound |  |
| --- | --- | --- |
| Analyte 1 | L-Tryptophan | IS 1 |
| Analyte 2 | L-Kynurenine | IS 2 |
| Analyte 3 | Kynurenic acid | IS 3 |
| Analyte 4 | Anthranilic acid | IS 4 |
| Analyte 5 | 3-Hydroxykynurenine | IS 10 |
| Analyte 6 | 3-Hydroxyanthranilic acid | IS 4 |
| Analyte 7 | Xanthurenic acid | IS 4 |
| Analyte 8 | Quinolinic acid | IS 2 |
| Analyte 9 | Picolinic acid | IS 10 |
| Analyte 10 | Nicotinic acid | IS 10 |
| Analyte 11 | *N*-Formylanthranilic acid | IS 10 |
| Analyte 12 | Cinnabarinic acid | IS 7 |
| Analyte 13 | Indole-3-acetic acid | IS 14 |
| IS 1 | Tryptophan-^2^H_5_ | - |
| IS 2 | Kynurenine-^2^H_4_ | - |
| IS 3 | Kynurenic acid-^2^H_5_ | - |
| IS 4 | Anthranilic acid-^2^H_4_ | - |
| IS 5 | 3-Hydroxyanthranilic acid-^2^H_3_ | *-* |
| IS 6 | Xanthurenic acid-^2^H_4_ | - |
| IS 7 | Quinolinic acid-^2^H_3_ | - |
| IS 8 | Picolinic acid-^2^H_3_ | - |
| IS 9 | Nicotinic acid-^2^H_4_ | - |
| IS 10 | Indole-3-acetic acid-^2^H_5_ | *-* |

1. Group 4–analyzed with Inertsil ODS-3

| Number | Compound |  |
| --- | --- | --- |
| Analyte 1 | Carnitine | IS 1 |
| Analyte 2 | Acetylcarnitine | IS 2 |
| Analyte 3 | Propionylcarnitine | IS 2 |
| Analyte 4 | Butyrylcarnitine | IS 2 |
| Analyte 5 | Pivaloylcarnitine | IS 2 |
| Analyte 6 | Hexanoylcarnitine | IS 2 |
| Analyte 7 | Octanoylcarnitine | IS 2 |
| Analyte 8 | Decanoylcarnitine | IS 2 |
| Analyte 9 | Lauroylcarnitine | IS 2 |
| Analyte 10 | Myristoylcarnitine | IS 2 |
| Analyte 11 | Palmitoylcarnitine | IS 2 |
| Analyte 12 | Stearoylcarnitine | IS 2 |
| IS 1 | DL-[^2^H_9_] carnitine hydrochloride | - |
| IS 2 | Hexanoyl-L-[^2^H_3_] carnitine | - |
| IS 3 | Stearoyl-L-[^2^H_3_] carnitine | - |

IS, internal standard

GSSG, glutathione oxidized form; GSH, glutathione reduced form.
