# Supplementary figures and images for: Metabolomic Analysis to Elucidate Mechanisms of Sunitinib Resistance in Renal Cell Carcinoma
Source: Metabolites. 2020 Dec 22;11(1):1. doi: 10.3390/metabo11010001 (PMC7821950; doi:10.3390/metabo11010001)

## Slide 1
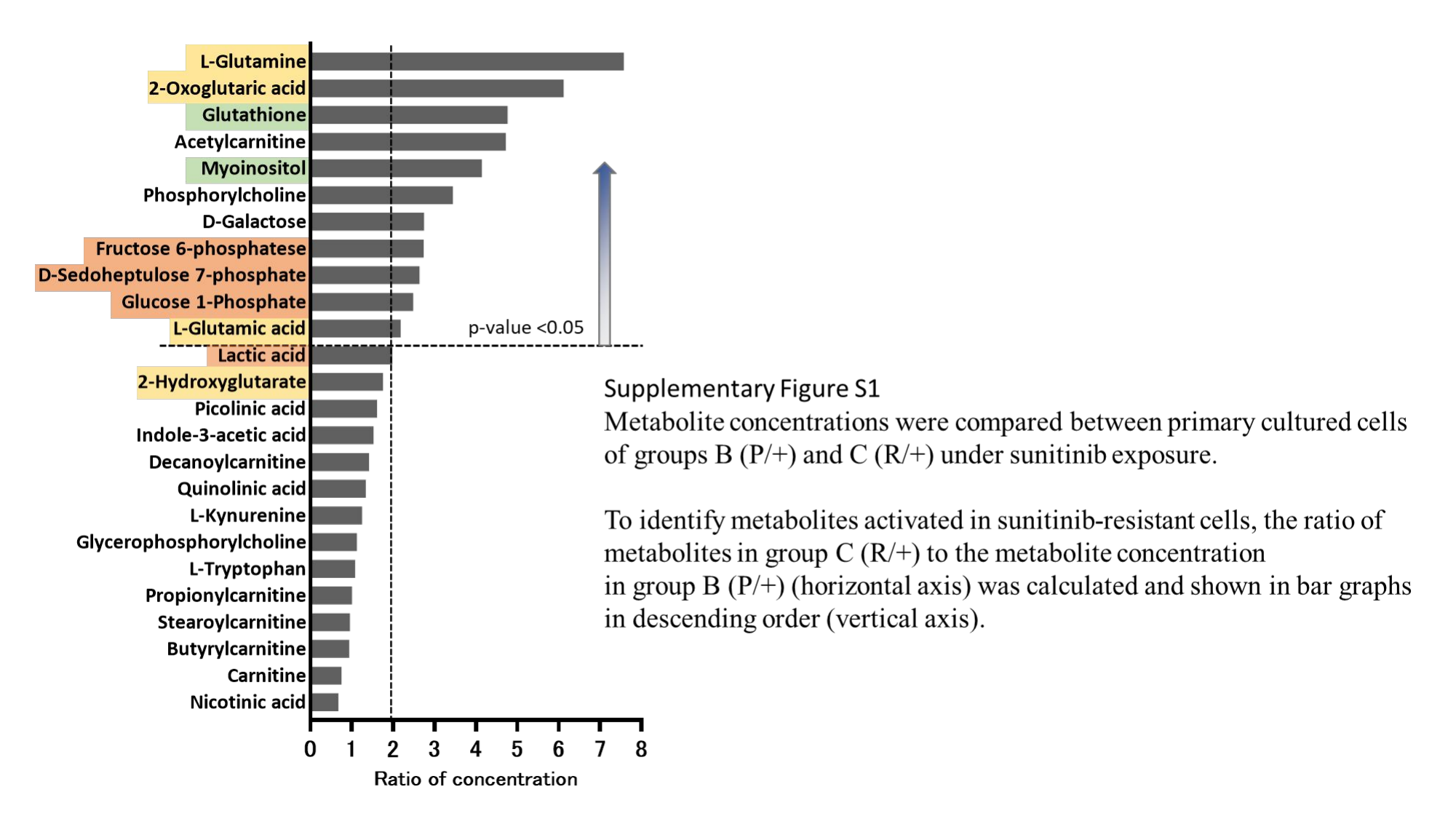

Supplement: Supplementary file 1 [file metabolites-11-00001-s001.zip › Supplementary/20201214Supplementary Figure S1.pptx]
